# Nourseothricin N-acetyl transferase (NAT), a new selectable marker for nuclear gene expression in *Chlamydomonas*

**DOI:** 10.1186/s13007-019-0526-5

**Published:** 2019-11-19

**Authors:** Xinjia Yang, Jialin Peng, Junmin Pan

**Affiliations:** 1 MOE Key Laboratory of Protein Sciences, Tsinghua-Peking Center for Life Sciences, School of Life Sciences, Tsinghua University, Beijing, 100084 China; 2 Laboratory for Marine Biology and Biotechnology, Qingdao National Laboratory for Marine Science and Technology, Qingdao, 266000 Shandong China

**Keywords:** *Chlamydomonas*, Nourseothricin N-acetyl transferase, Transformation, Selectable marker, Genetic engineering

## Abstract

**Background:**

*Chlamydomonas reinhardtii* is a unicellular green alga, which is a most commonly used model organism for basic research and biotechnological applications. Generation of transgenic strains, which usually requires selectable markers, is instrumental in such studies/applications. Compared to other organisms, the number of selectable markers is limited in this organism. Nourseothricin (NTC) N-acetyl transferase (NAT) has been reported as a selectable marker in a variety of organisms but not including *C. reinhardtii*. Thus, we investigated whether *NAT* was useful and effective for selection of transgenic strains in *C. reinhardtii*. The successful use of *NAT* would provide alterative choice for selectable markers in this organism and likely in other microalgae.

**Results:**

*C. reinhardtii* was sensitive to NTC at concentrations as low as 5 µg/ml. There was no cross-resistance to nourseothricin in strains that had been transformed with hygromycin B and/or paromomycin resistance genes. A codon-optimized *NAT* from *Streptomyces noursei* was synthesized and assembled into different expression vectors followed by transformation into *Chlamydomonas*. Around 500 transformants could be obtained by using 50 ng DNA on selection with 10 µg/ml NTC. The transformants exhibited normal growth rate and were stable at least for 10 months on conditions even without selection. We successfully tested that *NAT* could be used as a selectable marker for ectopic expression of *IFT54-*HA in strains with paromomycin and hygromycin B resistance markers. We further showed that the selection rate for *IFT54-*HA positive clones was greatly increased by fusing *IFT54-*HA to *NAT* and processing with the FMDV 2A peptide.

**Conclusions:**

This work represents the first demonstration of stable expression of *NAT* in the nuclear genome of *C. reinhardtii* and provides evidence that *NAT* can be used as an effective selectable marker for transgenic strains. It provides alterative choice for selectable markers in *C. reinhardtii*. *NAT* is compatible with paromomycin and hygromycin B resistance genes, which allows for multiple selections.

## Background

*Chlamydomonas reinhardtii* (*C. reinhardtii),* a unicellular green alga, is a widely used model organism for basic scientific research as well as biotechnological applications [1]. Generation of transgenic strains plays a critical role in our deeper understanding of molecular mechanisms involved in various cellular processes and genetic engineering for producing valuable products [2, 3].

Because of low efficiency of transformation, a selectable marker is usually needed for selection of transgenic strains. Currently, there are three types of selections used in nuclear transformation of *C. reinhardtii*: auxotrophy rescue, herbicide resistance and antibiotic resistance [1–3]. Auxotrophy rescue involves using parental strains with mutations thus limiting its application. For example, *Nit1*, a gene encoding nitrate reductase, can only be used in transformation of strains with defects in *Nit1* [4]. Several herbicide resistance markers have been reported [5–7]. The herbicides used include dichlorophenyl dimethyl urea (DCMU), norflurazon, oxyfluorfen, glyphosate and sulfadiazine. For reasons unknown, the herbicide resistance markers are rarely adopted in the community. It is likely due to high dose application of herbicide, poor transformation efficiency and/or other reasons. Six antibiotics have been used in *C. reinhardtii* for selection of transgenic strains transformed with corresponding selectable markers [1, 3]. The antibiotics used include paromomycin, zeocin, spectinomycin, hygromycin B, kanamycin and tetracycline. According to our understanding, only paromomycin, hygromycin B and zeocin resistance genes are commonly used as selectable markers [8–10]. Zeocin for selecting of *Ble* gene transformants is light sensitive and may induce genomic damages even in cells harboring the selection marker [11]. Compared to higher number of selectable markers in higher plant and mammalian cells [12, 13], the number of effective selectable markers is limited in *C. reinhardtii*. Therefore, availability of additional selectable markers in *C. reinhardtii* will enable complex experimental design, for example triple or more selection for transgenic strains.

Nourseothricin (NTC), a metabolite produced by *Streptomyces noursei*, belongs to streptothricin-class aminoglycoside antibiotics that inhibit protein synthesis [14]. NTC N-acetyl transferase (NAT) derived from *S. noursei* inactivates NTC by acetylating the beta-amino group of the beta-lysine residue [15]. NTC is highly soluble in water (1 g/ml) and stable for 2 years even in solution. *NAT* has been used as a selectable marker in a variety of organisms including bacteria, fungi, plant and mammalian cells (https://www.jenabioscience.com/images/741d0cd7d0/NTC-Flyer.pdf). However, *NAT* has been used in diatoms but not in other microalgae including *C. reinhardtii* [16].

In this report, we have shown that *NAT* is an effective selectable marker for nuclear transformation of *C. reinhardtii*. NTC, as low as 5 µg/ml, effectively kills or suppresses the growth of *C. reinhardtii* wild type cells as well as strains harboring paromomycin and/or hygromycin B resistant genes. Codon-optimized *NAT* from *S. noursei* is expressible in *C. reinhardtii* and confers cell resistance to NTC. We further show that *NAT* can be used as a selectable marker for transgenic strains even in strains harboring paromomycin and/or hygromycin B resistant genes. Furthermore, by fusing of a target gene to *NAT* and processing with the FMDV 2A peptides, the selection efficiency for targeted transgenic transformants is dramatically increased.

## Results

### Wild type *C. reinhardtii* strain is sensitive to nourseothricin

**Fig. 1:**
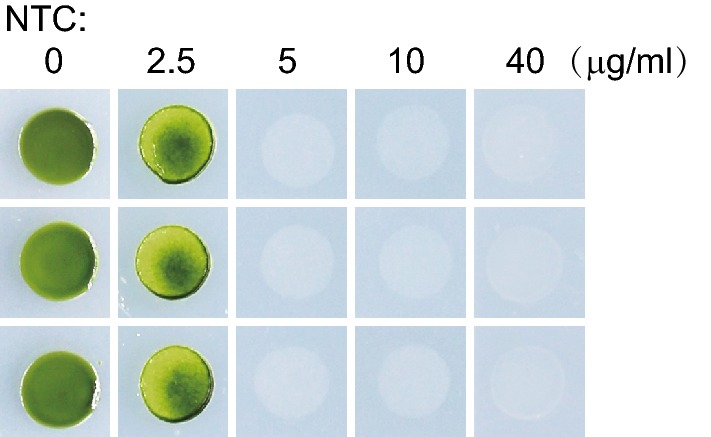
NTC efficiently kills or suppresses *C. reinhardtii* wild type cells. Wild-type cell cultures (1 × 10^6^ cells) were grown for 4 days on TAP agar plates supplemented with 0, 2.5, 5, 10, 40 µg/ml of NTC. Photos were taken after 4 days. Data shown are representative of three experiments

To explore the possibility to use *NAT* gene as a selectable marker for nuclear transformation, we first tested the sensitivities of *C. reinhardtii* to NTC. The selection concentrations for other organisms range from 20–400 µg/ml (https://www.jenabioscience.com/images/741d0cd7d0/NTC-Flyer.pdf). *C. reinhardtii* cells were placed on agar plates supplemented with different concentrations of NTC and grown for 4 days. The cells were sensitive to NTC at concentrations even as low as 2.5 µg/ml. At concentrations of 5 µg/ml and above, no viable cells were observed microscopically (Fig. 1) and even after 14 days (data not shown). Thus, we conclude that *C. reinhardtii* is sensitive to NTC, which paves the way for using *NAT* as a selectable maker.

### NTC is compatible with paromomycin and hygromycin B selections

For studies involved with transgenic strains, multiple selections are usually required. For example, rescue of insertional mutants generated by using antibiotic resistance genes requires another antibiotic selectable maker. Commonly used antibiotics for selection of transgenic strains of *C. reinhardtii* include paromomycin and hygromycin B [1]. We wondered whether NTC is compatible with these two antibiotics for selection in *C. reinhardtii*. NTC, paromomycin and hygromycin B all inhibit protein synthesis, but their working mechanisms are different. Paromomycin inhibits initiation of translation or earlier steps of elongation while hygromycin B potently inhibits elongation [17]. NTC inhibits protein synthesis with miscoding activity [14]. It has been shown in mammalian cells and fungus that NTC is compatible with selections with hygromycin B [18]. Whether it is compatible with selection with paromomycin and/or hygromycin B in *Chlamydomonas* cells is not known.

**Fig. 2:**
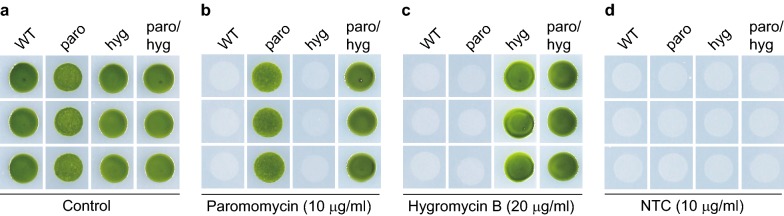
No cross-resistance to NTC in strains with paromomycin and/or hygromycin B resistance. Wild type (WT) cells and transgenic strains with resistance to paromomycin, hygromycin B or both were grown for 4 days on TAP agar plates without antibiotics (**a**) or supplemented with paromomycin (**b**), hygromycin B (**c**) or NTC (**d**). paro, paromomycin resistant strains; hygro, hygromycin B resistant strains; paro/hygro, paromomycin and hygromycin B double resistance strains. Data shown are representative of three experiments

Previously, our lab has generated strains with resistance to paromomycin, hygromycin B or both, which provided the needed resource for testing NTC compatibility with paromomycin and/or hygromycin B for selection. Wild type strain *21gr* and strains with paromomycin, hygromycin B or both were grown on agar plate supplemented the antibiotics as indicated. As shown in Fig. 2, application of NTC killed wild type cells as well as cells with paromomycin, hygromycin B and both. Thus, there is no cross-resistance to NTC in strains that had been transformed with hygromycin B and/or paromomycin resistance genes, indicating that NTC can be used for multiple selection with paromomycin and/or hygromycin B.

### Expression of codon optimized *NAT* gene confers resistance to NTC of *C. reinhardtii*

**Fig. 3:**
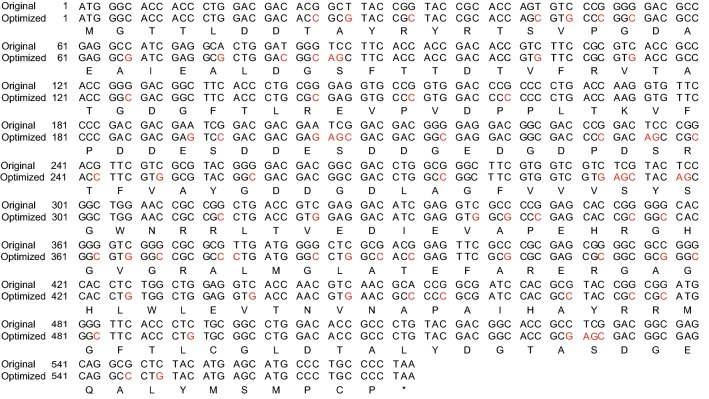
cDNA and amino acid sequences of *NAT* from *S. noursei* and codon optimization. Codon optimization was performed using OptimumGene™ algorithm. Optimized nucleotides are shown in red. Amino acid is indicated below each codon

**Fig. 4:**
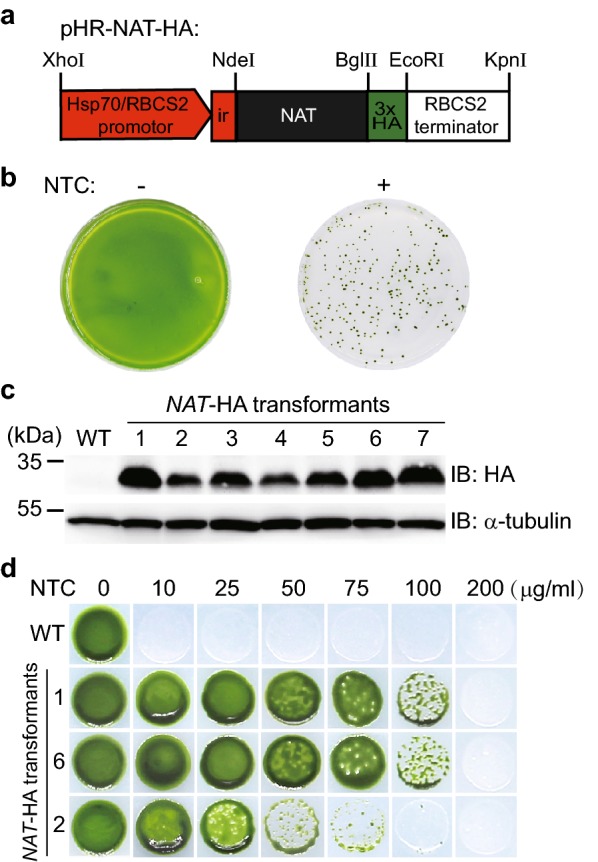
Expression of *NAT* confers cell resistance to NTC. **a** A diagram showing the construct for expressing *NAT* in *C. reinhardtii*. **b** WT cells were transformed with plasmid pHR-NAT-HA and grown on TAP agar plates supplemented with or without NTC (10 µg/ml). **c** Expression of *NAT*-HA was examined by immunoblotting with anti-HA antibody. α-tubulin was used as a loading control. **d** The extent of NTC resistance is correlated with the levels of *NAT* expression. Cells of wild type and strains with higher (#1 and #6) or lower (#2) expression levels of *NAT* were grown on agar plates supplemented with different concentrations of NTC as indicated

The *Chlamydomonas* genome has a higher GC content and its gene expression exhibits bias of codon usage. Divergence of these properties in a foreign gene may adversely affect gene expression in *Chlamydomonas* likely due to changes in chromatin structure and abnormal low abundance of some tRNAs, respectively [19–22]. Though the GC content of *NAT* is similar to that of *Chlamydomonas*, some rarely used codons in *Chlamydomonas* are present in *NAT*. To test whether *NAT* can be used a selectable marker, the *NAT* gene from *S. noursei* was synthesized with codon optimization (Fig. 3). The *NAT* gene encodes a protein of 190 aa. We generated a pHR-NAT-HA plasmid harboring codon optimized *NAT* (Fig. 4). The *NAT* ORF tagged with 3xHA was placed under the control of the *HSP70a/RBCS2* promoter containing one copy of *RBCS2* intron 1 and *RBCS2* 3′UTR (Fig. 4a). The construct was transformed by electroporation into wild type (WT) cells. The transformants were grown on agar plates with or without NTC (Fig. 4b). Transformants on agar plates without NTC grew like a lawn. In contrast, individual colonies were observed on agar plate with NTC, suggesting that these colonies are NTC resistant. The NTC resistance of these colonies may be caused by rare mutations or expressing *NAT*. To discern these possibilities, 7 colonies were randomly picked and subjected to analysis for *NAT* expression by immunoblotting. All the colonies showed expression of *NAT* (Fig. 4c). The protein mass of NAT was similar to calculated molecular weight. Thus, these data demonstrate that *NAT* can be successfully expressed in *C. reinhardtii* and its expression can confer transgenic strains with NTC resistance. The transformation efficiency was 528 cfu per 50 ng plasmid DNA for three independent transformations.

To examine the sensitivity of the transgenic strains with different levels of *NAT* expression, cells of strains with higher expression (strains #1 and #6) and lower expression (strain #2) were grown on agar plates supplemented with various concentrations of NTC (Fig. 4d). Consistent with results shown above, wild type cells were killed at 10 µg/ml NTC while the transformants grew normally. However, at higher concentrations of NTC, the transformants showed different extent of growth. For strain #2, which had lower expression of *NAT*, strong growth inhibition was observed at 50 µg/ml of NTC. In contrast, for strains #1 and #6, which had relatively higher expression of *NAT*, strong inhibition was observed at 100 µg/ml of NTC. These data further demonstrate that the tolerance to NTC is conferred by the expression of *NAT* and reveal that the extent of tolerance is correlated with the levels of *NAT* expression.

### *NAT* can be used as a selectable marker for transgenic strains

**Fig. 5:**
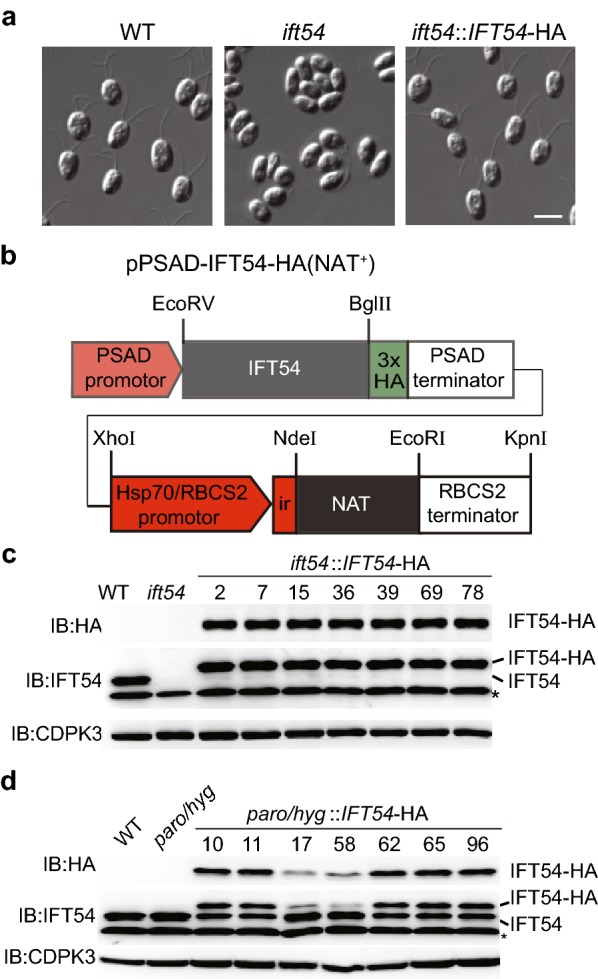
*NAT* as a selectable marker that is compatible with paromomycin and/or hygromycin B resistant genes. **a** Differential interference contrast (DIC) images showing wild type cells, *ift54* mutant and rescued cells. Please note that *ift54* did not have flagella and flagellar formation was rescued in *IFT54-*HA expressing cells. Bar, 10 μm. **b** A diagram showing construct that harbors expression cassettes of *IFT54*-HA and *NAT*. **c** Expression of *IFT54-*HA in strains harboring paromomycin resistant gene by using *NAT* as a selectable marker. The construct listed above was transformed into *ift54,* which harbors paromomycin resistant gene*.* The transformants were selected on agar plates supplemented with 10 µg/ml NTC. Cell lysates from randomly picked transformants were subjected to immunoblotting using the indicated antibodies. CDPK3 was used as a loading control. *Denotes non-specific bands. Wild type (WT) and *ift54* cells were used as control. **d** Expression of *IFT54-*HA in strains harboring paromomycin and hygromycin B resistant genes by using *NAT* as a selectable marker. *wdr92*::WDR92-YFP strains with both paromomycin and hygromycin B resistant genes were transformed. The expression of *IFT54-*HA from cells grown on selection plate was examined by immunoblotting. WT and *wdr92*::WDR92-YFP (paro/hygro) cells were used as control

Given that *NAT* can be expressed and confer NTC resistance in *C. reinhardtii*, we decided to examine it as a selectable marker for transgenic strains. *ift54* is a mutant defective in *IFT54*, which was generated by insertional mutagenesis with paromomycin resistance gene *AphVIII* [23]. Loss of IFT54 blocks cilia formation (Fig. 5a). To prove our hypothesis, we generated a plasmid carrying *NAT* resistant gene as well as protein expression cassette of *IFT54* (Fig. 5b). The plasmid was transformed into *ift54* and the transformants were selected on agar plates with 10 µg/ml NTC. 12 out of 192 (6.5%) colonies grown on NTC selection agar plates expressed *IFT54*-HA as examined by immunoblotting (Fig. 5c and data not shown). And all strains expressing *IFT54*-HA rescued the aflagellar phenotype of *ift54* (Fig. 5a). These data demonstrate that *NAT* can be used as a selectable marker even in strains with paromomycin resistance.

Next, we examined whether *NAT* can be used as a selectable marker in strains with both paromomycin and hygromycin B resistance. The pPSAD-IFT54-HA(NAT^+^) was transformed into a strain with paromomycin and hygromycin B resistance. 7 out of 116 (6.03%) colonies grown on the NTC selection plates expressed *IFT54*-HA as examined by immunoblotting (Fig. 5d and data not shown). Taken together, we have shown that *NAT* is an efficient selectable marker, which is compatible with paromomycin and/or hygromycin B resistance genes.

### Fusion of a target gene *IFT54-*HA to *NAT* and processing with the FMDV 2A peptide increases gene expression efficiency

**Fig. 6:**
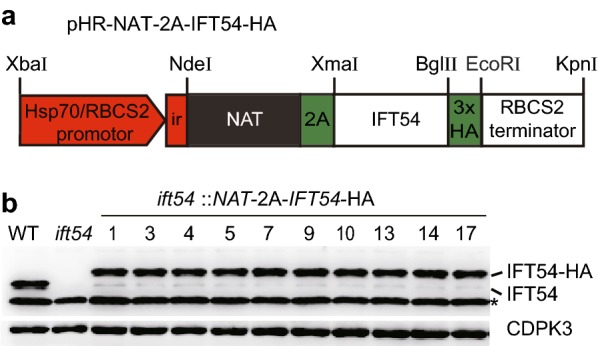
Fusion of *IFT54-*HA to *NAT* and FMDV 2A for cell transformation. **a** Schematic representation of the construct used to drive *NAT-*2A*-IFT54-*HA fusion protein expression. **b** Immunoblot analysis of *IFT54-*HA expression in transformants from the fusion construct mediated transformation. Cell lysates from WT, *ift54* and the transformants were subjected to immunoblotting with IFT54 and CDPK3 antibodies. CDPK3 was used as a loading control. *Denotes a band in each lane that reacts non-specifically. Please note, the predicted fusion protein NAT-FMDV 2A–IFT54-HA was cleaved in the cell to generate IFT54-HA

It has been reported that the gene expression efficiency is much improved by fusion of a target gene to a selectable marker and processing with the FMDV 2A peptide [24]. Due to cleavage after gene translation at the 2A peptide sequence, the resulting protein is processed into two discrete proteins: a protein from the target gene and the selectable marker protein fused with short 2A peptide [25]. To demonstrate whether *NAT* can be used as such a selectable marker, we made a construct by fusing *IFT54-*HA to *NAT* and DNA sequence of FMDV 2A (Fig. 6a). The construct was transformed into *ift54* and the transformants were selected on agar plates with 10 µg/ml NTC. 122 out of 204 (59.8%) colonies had flagella. Examination of a few transformants with flagella showed that they all expressed *IFT54*-HA (Fig. 6b). Thus, we predicted that all the transformants that had formed flagella should have expressed *IFT54*-HA. Compared to the construct that does not fuse *IFT54-*HA to *NAT* as shown in Fig. 5b*,* this construct led to a ninefold increase of selection efficiency (6.5% to 59.8%) for transgenic strains. However, by examination of the protein levels of IFT54-HA in transformants derived from these two constructs (Figs. 5c and 6b), we have not observed increased protein expression level by such a fusion construct as reported [24].

## Discussion

The ability to generate transgenic cells is crucial for genetic engineering widely used in basic research as well as in biotechnological applications. As a model organism, *C. reinhardtii* is widely used for exploration of basic cellular processes such as cilia biogenesis and photosynthesis and for producing commercially valuable products as a cell factory [1–3]. Although a few selectable markers have been developed in this organism, few of them have been widely used. An ideal selectable marker may possess the following properties: (1) high stability, aqueous solubility and low dosage of the selection reagents; (2) non-toxicity of the selection reagents in the presence of a selectable marker; (3) non-toxicity of the selectable markers; 4) high efficiency of transformation; (5) compatibility with other selectable makers and 6) no genotype requirement for the parental strains.

We have shown that *NAT* is an effective and stable selectable marker in *C. reinhardtii* that confers resistance to NTC. *C. reinhardtii* is very sensitive to NTC. No viable colonies were observed even in the presence of 5 µg/ml NTC though we have used 10 µg/ml for the selection. NTC is soluble in water and highly stable. The transformation efficiency of *NAT* is high. Around 500 cfu were routinely obtained by using 50 ng plasmid DNA for transformation. Expression of *NAT* was stable even in the absence of NTC. We have not observed any growth defects in *NAT* transgenic strains. As NTC is an antibiotic, it does not require strains with specific genotype. Thus, *NAT* provides an alternative choice for selectable markers in *C. reinhardtii*. Random insertion of foreign DNA into the genome of *C. reinhardtii* occurs during transformation and this property has been used to generate insertional mutants [26, 27]. Though we have not examined the patterns of integration of *NAT* into the genome of *C. reinhardtii*, *NAT* is expected to behave as other foreign DNA fragments. Thus, *NAT* may be used for generation of insertional mutants from which desired functional genes can be cloned.

We have tested using *NAT* as a selectable marker for transgenic expression of a target gene *IFT54*. We have used parental strains that had been previously transformed with paromomycin and/or hygromycin B resistant genes. Around 6.5% *IFT54* transgenic strains were obtained from *NTC* resistant colonies, demonstrating that *NAT* can be used as a selectable marker. These data also indicate that *NAT* is compatible with hygromycin B and paromomycin resistant genes, which allow for multiple selections. We have developed a construct by fusing the target gene *IFT54* to *NAT* and processing with FMDV 2A peptide. Compared to the non-fusion construct, the efficiency of expression of *IFT54*-HA has increased around ninefold. Thus, this fusion expression system can increase selection efficiency of transgenic strains. Because the NTC resistance is correlated with the expression levels of *NAT*, this system may also be used for obtaining strains with higher expression of target genes by selection at higher concentrations of NTC.

*NAT* as a selectable marker has been used in microalgae but so far only in marine diatoms including *Chaetoceros gracilis*, *P. tricornutum* and *T. pseudona*na [16, 28, 29]. Our demonstration that *NAT* can be used a selectable marker in a fresh water green alga, opening a promising prospect in using *NAT* in other microalgae, especially in those algae with fewer choices for selectable markers. For example, *Dunaliella*, a saline green alga, is a popular model organism for the study of adaptation of eukaryotic cells to high salt concentrations and some *Dunaliella* species are of economic value for producing beta-carotene [30]. However, *Dunaliella* is resistant to paromomycin, hygromycin B, spectinomycin and kanamycin [3]. Thus, NTC resistance needs to be tested in *Dunaliella* before the NAT/NTC selection system can be used.

## Conclusions

We have developed a new stable selectable marker for selection of transgenic strains in *C. reinhardtii* that confers resistance to NTC, which provides an alternative choice for selectable markers. In addition, *NAT* is compatible with paromomycin and hygromycin B resistance genes, two most commonly used selectable markers in *C. reinhardtii*, which allows combination of multiple selectable markers in transgenic studies.

## Methods

### Strains and culture

*Chlamydomonas reinhardtii* wild type strain *21gr* (CC-1690, mt+) was from the *Chlamydomonas* Resource Center. *ift54* (a paromomycin resistant strain) [23], *lf4*::LF4-HA (a hygromycin B resistant strain) [31] and *wdr92*::WDR92-YFP (a paromomycin and hygromycin B double resistant strain) [32] were generated in our own lab. Unless otherwise stated, cells were grown at 23 °C in M liquid medium in a 14/10 light/dark cycle [33]. Cells used for transformation were grown at 23 °C in liquid TAP medium under continuous light [34].

### Reagents

Paromomycin and hygromycin B were purchased from Merck Millipore, USA, while NTC was obtained from Jena biosciences, Germany. The antibiotics were solubilized in water and sterilized by filtration. The concentrations used for selection for paromomycin, hygromycin B and NTC were 10, 20 and 10 µg/ml, respectively.

### Drug sensitivity assay

To determine the sensitivity of *C. reinhardtii* to NTC, 1 × 10^6^ of wild type cells were spotted on 1.5% TAP agar plates supplemented with various concentrations of NTC (0, 2.5, 5, 10 and 40 µg/ml) and incubated for 4 days at 23 °C in a 14/10 light/dark cycle. To test whether strains with paromomycin and/or hygromycin B resistant genes are sensitive to NTC, 1 × 10^6^ cells of wild type and strains with various resistant genes were grown on 1.5% TAP agar plates supplemented with different antibiotics as indicated in the text.

### Construction of the transformation vectors

To generate a construct for expressing *NAT*, the coding region of *NAT* from *S. noursei* was codon optimized for *C. reinhardtii* and chemically synthesized (Genscript, China). Codon optimized *NAT* tagged with 3× HA tag at the 3′ end driven by *HSP70a/RBCS2* and terminated by *RBCS2* terminator was cloned into ZT4-blunt vector. The *HSP70a/RBCS2* promoter and *RBCS2* terminator were cloned from pCB740 [35]. The 3× HA tag was cloned from pKL-3XHA [36]. The final construct was termed pHR-NAT-HA. To enable expression of *IFT54-*HA in *C. reinhardtii* with *NAT* as a selectable marker, the expression cassette for hygromycin B in the vector pPSAD-IFT54-HA-Hyg was replaced with the *NAT* expression cassette in pHR-NAT-HA with 3xHA being removed [23]. The resulting construct was termed pPSAD-IFT54-HA(NAT^+^). To generate construct pHR-NAT-2A-IFT54-HA for fusion of *IFT54-*HA to *NAT* and processing by FMDV 2A peptide, the sequence for *Ble* and *GFP-tubulin* in the vector pBR25-sfGFP-TUA were replaced by *NAT* and *IFT54-*HA, respectively [37]. All the constructs were verified by sequencing.

### Electroporation transformation

Transformation of *Chlamydomonas* was performed by electroporation using BTX ECM630 (Harvard Apparatus Inc, USA) following a previously published protocol [38]. For each transformation, 5 × 10^7^ cells were mixed with 50 ng plasmid DNA linearized by *AclI*. After electroporation, the transformation mixture was diluted in 10 ml TAP + 50 mM sorbitol and kept away from light for 8 h. Transformants were selected on agar plates supplemented with 10 µg/ml NTC.

### SDS-PAGE and immunoblotting

SDS-PAGE and immunoblotting analysis were performed as described previously [39]. Briefly, cells were lysed with Buffer A (50 mM Tris–HCl pH 7.5, 10 mM MgCl_2_, 1 mM EDTA, and 1 mM DTT) containing protease inhibitor cocktail (Roche, Switzerland) and boiled for 10 min in 1× SDS loading buffer. The proteins were separated in 10% SDS-PAGE, transferred to polyvinylidene difluoride (PVDF) membranes (Merck Millipore, USA) and probed with the indicated antibodies. The primary antibodies used include the following: rat anti-HA (Roche, Switzerland), 1:3000; mouse anti-α-tubulin (Sigma-Aldrich, USA), 1: 3000; rabbit anti-IFT54, 1:3000 [23] and rabbit anti-CDPK3, 1: 5000 [38].

### Cell imaging

After cell fixation in 1% glutaraldehyde, DIC images were captured by Zeiss Axio Observer Z1 microscope (Carl Zeiss, Germany) equipped with a CCD camera (QuantEM512SC, Photometrics, USA). The images were processed in Photoshop and/or Illustrator (Adobe, USA).

## Data Availability

All data generated or analyzed during this study are included in this published article. Experimental materials generated during the current study are available from the corresponding author on reasonable request.

## Abbreviations

NAT: nourseothricin (NTC) N-acetyl transferase
NTC: nourseothricin
FMDV 2A: foot-and-mouth-disease-virus 2A
HR: hSP70a/RBCS2
IFT54: intraflagellar transport 54
CDPK3: calcium dependent kinase 3
DIC: differential interference contrast
